# Role of Intravenous Levetiracetam in Seizure Prophylaxis of Severe Traumatic Brain Injury Patients

**DOI:** 10.3389/fneur.2013.00170

**Published:** 2013-11-01

**Authors:** Batool F. Kirmani, Diana Mungall, Geoffrey Ling

**Affiliations:** ^1^Department of Neurology, Epilepsy Center, Scott & White Neuroscience Institute, Temple, TX, USA; ^2^Texas A&M Health Science Center College of Medicine, Temple, TX, USA; ^3^Uniformed Services University of Health Sciences, Bethesda, MD, USA

**Keywords:** levetiracetam, seizure prophylaxis, traumatic brain injury, phenytoin, epilepsy

## Abstract

Traumatic brain injury (TBI) can cause seizures and the development of epilepsy. The incidence of seizures varies from 21% in patients with severe brain injuries to 50% in patients with war-related penetrating TBI. In the acute and sub-acute periods following injury, seizures can lead to increased intracranial pressure and cerebral edema, further complicating TBI management. Anticonvulsants can be used for seizure prophylaxis according to the current Parameters of Practice and Guidelines in a subset of severe TBI patients, and for a limited time window. Phenytoin is the most widely prescribed anticonvulsant in these patients. Intravenous levetiracetam, made available in 2006, is now being considered as a viable option in acute care settings if phenytoin is unavailable or not feasible due to side-effects. We discuss current data regarding the role of intravenous levetiracetam in seizure prophylaxis of severe TBI patients and the need for future studies.

## Introduction

### Post traumatic seizures

The annual incidence of traumatic brain injury (TBI) is 1.7 million in the United States (1). One well-recognized complication of moderate to severe TBI is post traumatic seizure (PTS). A study by Temkin et al. shows a 2-year seizure rate of 21% after severe TBI (2). In another study by Salazar et al. the incidence of seizures is almost 50% after penetrating war-related TBI (3). It is generally accepted that mild TBI or concussion can increase the risk of developing epilepsy but the incidence is uncertain. This uncertainty is largely due to incomplete knowledge of the incidence of mild TBI itself.

Post traumatic seizure are divided into two subgroups. The seizures occurring within the first 7 days after brain injury are classified as early PTS. Those occurring after 7 days of injury are classified as late PTS. Non-convulsive electrographic seizures can also occur. These can lead to cerebral metabolic crisis, delayed increase in intracranial pressure, and worse clinical outcome (4).

The deleterious effects of PTS exacerbate the existing brain injury and contributes to greatly worse TBI outcome. Consequently, an important clinical goal in TBI care is preventing seizures. To this end, Temkin et al. conducted a series of randomized placebo controlled trials (RCT) that demonstrated efficacy of phenytoin, valproate, and carbamazepine in reducing the incidence of early PTS (2, 5, 6). These same studies showed that there was no benefit for late PTS.

Phenytoin is the preferred agent of choice for early PTS prophylaxis. It is generally well tolerated, can be administered once per day, can be given IV and most medical practitioners are familiar with its use. Valproate is less desirable as it is shown to be associated with increased mortality. Carbamazepine is not yet clinically available in an IV formulation. This issue is important because most moderate to severe TBI patients cannot take medications orally due to inability to protect their airways. Thus, valproate and carbamazepine should be considered as alternatives to phenytoin for early PTS prophylaxis (7, 8).

Phenytoin has significant adverse effects. The most common are hypersensitivity reactions, irritation of the skin, phlebitis, arrhythmias, and hypotension during parenteral administration (9). One particularly severe toxicity is Stevens Johnson Syndrome. Phenytoin has a narrow therapeutic index and non-linear kinetics so a small increase in dose may result in much greater increase in levels resulting in toxicity. Additionally, it is more prone to drug–drug interactions because of the induction of the hepatic cytochrome P450 system which further limits its use in critically ill patients (10). Phenytoin has also been shown to exacerbate acute adrenal hyporesponsiveness, a phenomenon seen in patients with severe brain injury by decreasing the cortisol concentration (11–14). A limitation frequently encountered with phenytoin is the use of weight based dosing, which often leads to subtherapeutic levels of the drug (15).

Because of these complications and the limitations of valproate and carbamazepine, levetiracetam is more often being considered as a viable option.

Intravenous levetiracetam was approved by the FDA in 2006 and has a number of advantages over phenytoin. Levetiracetam has linear pharmacokinetics (PKs) and is thus easier to titrate. It has lower potential of drug–drug interaction than phenytoin. It has not yet been shown to have enzyme inducing properties. Finally, there is no need to monitor of serum drug levels (16). In this review, we will discuss the studies which provide evidence of efficacy of intravenous levetiracetam in the prevention of PTS.

Preclinical studies using animal models of TBI have shown efficacy of levetiracetam as a neuroprotectant. Zou et al. report a study of rats treated with levetiracetam after receiving a controlled cortical impact (CCI) TBI. In this study, 50 mg/kg of either intraperitoneal levetiracetam or saline control were administered daily for 20 days starting 1 day after CCI or sham injury. To determine neurobehavior outcome, rats were tested on balance beam, Y-maze, and the Morris Water Maze. Levetiracetam treatment was shown to be beneficial to neurobehavior functional recovery and hippocampal cell sparing. It also decreased contusion volumes by almost 33%. Finally, TBI-induced decreases in regional glutamate transporter expression and neuroplastic markers were reversed by levetiracetam. The investigators concluded that levetiracetam treatment post-TBI, lead to improved histological, molecular, and neurobehavioral outcomes (17).

In spite of these promising preclinical studies, levetiracetam has not yet been conclusively shown to have neuroprotective or neuro rescue effects in humans.

However, there is human clinical evidence to show that levetiracetam is a reasonable alternative to phenytoin for seizure prophylaxis. In a prospective multicenter comparison of levetiracetam vs. phenytoin for early PTS prophylaxis by Inaba and colleagues, patients with closed head TBI are treated with either medication. A total of 813 patients are analyzed of which 406 received levetiracetam and 407 received phenytoin. The two groups are balanced for age, gender, Injury Severity Score (ISS), Marshall score of ≥3 or craniectomy. There results reveal no statistically significant difference in terms seizure rate, adverse drug reactions, or mortality. The authors conclude that levetiracetam did as well but not better than phenytoin as an early PTS prophylaxis. The cost of Levetiracetam is higher than phenytoin and availability is also an issue. However, the need and cost of monitoring favors Levetiracetam (18).

For other than TBI brain pathological states, Zafar et al. report a meta-analysis of studies that compare levetiracetam to phenytoin for seizure prophylaxis for patients who suffer from TBI, intracranial hemorrhage, intracranial neoplasms, and/or craniotomy. A comprehensive electronic data search is performed on studies with a primary outcome of seizures and have balanced the baseline population characteristics, type of intervention, and study design. Of 2489 studies, 8 meet inclusion criteria. Of these, two are RCT and six observational studies. The results show odds ratio equal to 1.12 so there is no superiority of either agent in the prevention of early seizures. The conclusion of the meta-analysis is that levetiracetam and phenytoin have equal efficacy in seizure prevention after TBI as well as intracranial hemorrhage, intracranial neoplasm, and craniotomy. The limitation of this study is that the conclusions are based on a few RCT. Thus, additional trials are recommended (19).

Szaflarski et al. describe the first prospective randomized comparative trial of intravenous levetiracetam vs. phenytoin for seizure prophylaxis in the neuro intensive care unit setting. Fifty-two neuro intensive care unit patients are enrolled in this study. Many (89%) have severe TBI. Thirty-four patients receive levetiracetam and 18 phenytoin. Standard intravenous doses are used with doses of phenytoin adjusted to maintain therapeutic serum levels. Continuous EEG monitoring is performed during first 72 h of treatment. After controlling the baseline severity, there are better outcomes with levetiracetam therapy as evidenced by lower disability rating scales at 3 months and higher Glasgow outcomes scales at 6 months. No differences in occurrence of seizures are seen in either group during or at 6 months. No differences are seen in adverse effects in either group except for lower incidence of worsened neurological status and fewer gastrointestinal problems in the levetiracetam treated group. The authors conclude that levetiracetam is a reasonable alternative to phenytoin for seizure prophylaxis in the neuroscience ICU setting. The limitations of this study include a small sample size, and a lack of reported data on the use of sedating agents in these patients. Propofol is the drug of choice in patients with severe TBI, which also has anticonvulsant properties. The concomitant use of both agents may have confounded the results of the trial (20).

A retrospective, observational study is conducted by Kruer et al. to evaluate patients treated with phenytoin vs. levetiracetam for seizure development within 7 days after TBI. Of 1,552 TBI patients identified through the adult trauma center registry, 354 met inclusion criteria of ≥3 on the Abbreviated Injury Scale (AIS), and <8 on the Glasgow coma scale. A total of 245 patients are excluded, mostly because no prophylactic antiepileptic drug was given (34%) or there was no history of acute TBI (30%). The remaining 109 adults who received phenytoin (*N* = 89) or levetiracetam (*N* = 20) have additional information gathered from the electronic medical record and paper chart. One patient in each group had documented post-traumatic seizures. Sixty-five percent of patients received prophylactic AEDs for >7 days, and 68/109 patients survived to hospital discharge. Between 2000 and 2007, nearly all of the patients (98%) received phenytoin. This was reversed during 2008–2012 such that the majority (64%) patients instead received levetiracetam. Thus, after FDA approval of its IV formulation, a clinical trend favoring levetiracetam use over phenytoin is observed but the lack of difference between these drugs is due to the restricted results on PTS (21).

To determine efficacy for early PTS prophylaxis following severe TBI, Jones et al. compare levetiracetam vs. phenytoin. The study is conducted in 32 patients, all of whom receive IV levetiracetam for the first 7 days after TBI. These data are compared to results from a historical cohort of 41 patients who received phenytoin for seizure prophylaxis. Patients are evaluated for 1 h by EEG if they have persistent coma, decreased mental status, or clinical signs of seizures. The results show that 15/32 patients in the levetiracetam group and 12/41 in the phenytoin group require EEG monitoring. Of the levetiracetam group, the EEG from seven patients were normal and eight were abnormal. Of the EEG abnormal subgroup, one patient had EEG evident seizure activity and the rest had signs of seizure tendency but no overt evidence of seizure. The EEG results were all normal in phenytoin treated patients. For seizure activity, there was no statistical difference between these two treatment groups. The authors conclude that levetiracetam is as effective as phenytoin in preventing early clinical PTS, but a higher incidence of epileptogenicity with levetiracetam. The limitations of this study included lack of randomization, small sample size, and duration of the EEG recording. The rationale was that only 1-h recordings would not be able to capture actual seizures for which more prolonged monitoring is required (22).

Studies have shown that the PK profile of intravenous levetiracetam, which includes linear kinetics and lack of drug–drug interactions, favors this new agent over phenytoin. However, the PK properties of levetiracetam are not etiology specific and more studies are required in this subset of patients.

In a prospective, open-label, steady-state PK study by Spencer and colleagues, steady-state PKs of IV LEV is assessed in neurocritical care patients. The sample size of 12 adults, comprised of five men and seven women aged 54 ± 14 years, all required anticonvulsant prophylaxis in the neurocritical care unit after TBI (*N* = 1), subarachnoid hemorrhage (*N* = 10), or subdural hematoma (*N* = 1). Patients were eligible if they were >18 years old, presence of arterial or central venous access for blood sampling, and required IV LEV for seizure prophylaxis. Patients were excluded if they had multisystem trauma, end-stage renal disease, or hemoglobin concentration <7.0 g/dl. An IV infusion of 500 mg LEV given over 15 min every 12 h was administered to patients. After a minimum of four doses of LEV, serial blood samples were collected from all patients. Ultraperformance liquid chromatography with tandem mass spectrometry detection was used to determine the serum levetiracetam concentration. LEV maximum serum concentration was found to be a mean ± SD of 28.0 ± 8.0 μg/ml, minimum serum concentration 3.1 ± 1.8 μg/ml, and half-life 5.2 ± 1.2 h. The systemic clearance was 5.6 ± 1.8 l/h and the volume of distribution at steady state 36.8 ± 6.3l. The probability of achieving a target trough concentration of 6 μg/ml or greater was increased by greater doses of LEV, but it also increased the probability of reaching trough concentration greater than 20 μg/ml. The highest probability of achieving a target trough concentration of 6–20 μg/ml was when 1,000 mg of LEV every 8 h and 1,500–2,000 mg every 12 h. The authors concluded that the LEV systemic clearance was faster and the terminal elimination half-life was shorter in neurocritical care patients than in previously reported results in adults in status epilepticus or healthy volunteers. The study limitation is the sample size (23).

Klein et al. conducts a fixed dose, open-label, non-randomized, phase II safety, and PK study of patients, including children ≥6 years old, treated with levetiracetam after TBI with a high risk of post-traumatic epilepsy. A total of 26 children and 15 adults are enrolled, whose ages range from 6 to 87 years. TBI inclusion criteria are any intracranial hemorrhage, except for isolated subarachnoid hemorrhage or with penetrating wound injury, depressed skull fracture, or early PTS. Beginning ≤8 h after injury and lasting for a duration of 30 days, all subjects receive levetiracetam 55 mg/kg/day orally, nasogastrically, or intravenously. The initial dose is followed by two divided doses every 12 h for the study duration. On treatment days 3 and 30, all 41 subjects undergo PKs analysis. Thirty-six of 41 subjects are randomized to undergo PK study on treatment day 3, and 24/41 subjects are randomized to undergo PK study on day 30. Mean *T*_max_ on day 3 is 2.2 h, *C*_max_ was 60.2 μg/ml and area under the curve (AUC) is 403.7 μg/h/ml. *T*_max_ is shorter in children than in adults and elderly subjects (respectively, 1.5 and 1.8 vs. 5.96 h; *p* = 0.0001). Compared with adults and the elderly, the AUC is non-significantly lower in children (461.4 and 450.2 vs. 317.4 μg/h/ml). *C*_max_ is non-significantly higher after administration IV (0.4 μg/ml) vs. tablet (59 μg/ml) or NG (48.2 μg/ml). AUC of IV and NG administration is 88 and 79% of the AUC of oral administrations. Between days 3 and 30, the PKs are not significantly different. The authors conclude that TBI study subjects with a high PTS risk are treated with the same dose with antiepileptogenic effect in animals (55 mg/kg/day) achieve plasma LEV levels comparable to those in animal studies (24).

## Future Directions

The available data have shown limitations and one cannot clearly establish the efficacy and tolerability of intravenous levetiracetam over phenytoin in early seizure prevention in severe TBI. Phenytoin still remains the drug of choice based on the existing evidence and wide availability of PHT titration makes it easy to be managed safely. However, based on the existing data, levetiracetam seems to be a favorable choice for early seizure prophylaxis in patients with severe TBI. Levetiracetam can be used in situations where there is risk of drug–drug interactions and drug toxicity because of the narrow therapeutic index of phenytoin. However, further larger, prospective, randomized double blind multicenter trials are needed to further define the role of this anticonvulsant in short and long term seizure prophylaxis in patients with severe TBI.

## Conflict of Interest Statement

The authors declare that the research was conducted in the absence of any commercial or financial relationships that could be construed as a potential conflict of interest.
